# Effects of Repair Interface Structure on Mechanical Properties of Scarf Repair for Composite Laminate Plates

**DOI:** 10.3390/polym17040511

**Published:** 2025-02-16

**Authors:** Kaomin Zhang, Zhenhu Miao, Haiting Xia, Xiaoyu Yang, Fenglin Tian, Yinghui Zhao

**Affiliations:** 1 Faculty of Civil Aviation and Aeronautics, Kunming University of Science and Technology, Kunming 650500, China; zkmbuaa@163.com (K.Z.); 20222245014@stu.kust.edu.cn (X.Y.); tianfenglin23@163.com (F.T.); zyh_kust@163.com (Y.Z.); 2 Yunnan Low-Altitude Economy and Drone Technology Innovation Center, Kunming 650500, China

**Keywords:** composite materials, scarf repair, mixed scarf, adhesive interface, mechanical properties

## Abstract

The structural damage repair of composite material is an important issue that needs to be addressed during the service life of composite materials. To investigate the effects of a scarf structure and scarf angle on the repair quality of composite material, this paper proposes a mixed-scarf (MS) repair structure that combines ramped-scarf (RS) and stepped-scarf (SS) repair structures. The effect of the repair structure on the mechanical properties was analyzed, as well as the quality of the adhesive interface. The results show that at a scarf angle of 10°, the repair efficiency and the quality of adhesive interface are better than that of scarf angles of 20° and 30°. At a scarf angle of 10°, the recovery degree of the flexural strength of the MS repair structure is 79.72%, which is 6.77% and 38.24% higher than that of the RS and SS repair structures, respectively. However, in terms of flexural modulus, regardless of repair structure, the flexural modulus is highest at a scarf angle of 20°. Furthermore, the impact strength of the MS repair structure is approximately 87.60% that of the RS repair structure; additionally, it exhibits an increase of 45.83% compared to the SS repair structure. Overall, the quality of the adhesive interface for the RS and MS repair structures is similar and better than that of the SS repair structure. In conclusion, the MS repair structure is well suited for small-angle scarf repairs, whereas the RS repair structure is more appropriate for large-angle repairs; in contrast, the SS repair structure demonstrates the least effective performance in terms of repair outcomes.

## 1. Introduction

Advanced composite materials boast qualities such as lightness, high strength, corrosion resistance, fatigue resistance, and flexible design [1,2,3]. These materials have found widespread applications in aerospace, automotive, shipbuilding, and other sectors. The maintainability of advanced composite material structures stands as a crucial metric for assessing their operational efficiency, and it serves as a significant approach to minimize the full life-cycle costs of these structures. The primary methods for repairing composite material damage are patch-adhesive and scarf-adhesive repair [4,5]. Notably, scarf-adhesive repair offers several advantages, including high repair strength, minimal disruption to the surface aerodynamic shape, minimal weight increase, and excellent restoration of the structural integrity [6]; it is predominantly employed for repairing advanced composite material. The scarf structure and the bonding interface are crucial to the repair quality, as it directly impacts the repair strength of the bonding interface. There are two prevalent types of scarf structures: ramped scarf (RS) and stepped scarf (SS). RS repair structures involve processing damaged areas in a slanted manner, demanding high-end repair equipment and posing challenges for patch placement. Nevertheless, the quality of the bonding interface is superior, resulting in an effective repair quality. On the other hand, SS repair involves processing damaged areas in a stepped fashion. Compared to the RS repair structure, the SS repair structure offers simpler operation and easier patch preparation. However, the bonding interface may be prone to stress concentration, decreasing the repair efficiency [7,8,9].

In recent years, numerous scholars have undertaken comprehensive research on the adhesion processes involved in scarf repair. Pitanga et al. [10] utilized segmented ramps with different slopes for scarf repair and found that a repair structure with a slope of 1:30/1:2 performed the best. Marques et al. [11] repaired composite material laminates utilizing a ramped-scarf joint and conducted tensile tests to investigate the morphology and failure modes. Their findings indicate that the failure of the repair interface is the predominant failure mode in scarf repair structures. Bendemra et al. [7] developed ramped and stepped adhesive joints, investigating the influence of joint parameters on scarf repair structures. Karaduman et al. [12] conducted a comprehensive investigation into the effects of various repair methods and scarf angles on the mechanical performance of composite materials. Additionally, they compared single-scarf repairs with double-scarf repairs to identify a more robust solution. Wang et al. [13] propose a parameterized simulation framework designed to predict the load-bearing capacity of 3D scarf-repaired composite laminates. This innovative approach significantly reduces modeling time from several hours to merely a few seconds. Zhao et al. [2] conducted a reliability analysis of composite laminate patch-repaired structures subjected to unidirectional static loads using a probabilistic model. Xiao et al. [14,15] conducted a comprehensive study on the compressive failure behavior of stepped-scarf repair in composite stiffened panels through both experimental and simulation approaches. Their research elucidated the damage evolution within the composite and adhesive layers, while also investigating the effectiveness of stepped-scarf repair on honeycomb sandwich panels subjected to impact damage. Orsatelli et al. [16] developed finite element models to predict the stress distribution and strength of stepped repairs in composite materials. Damghani et al. [17,18] investigated the buckling and post-buckling performance of ramped and stepped repair structures under in-plane shear loads, showing that ramped repair structures outperform stepped repair structures in critical mechanical properties, and the failure modes of both types of repair structures are similar. Chen et al. [19] utilized experimental and simulation techniques to analyze the mechanical performance of ramped-scarf repairs in carbon fiber-reinforced composite laminates under low-speed impact, finding that ramped-scarf repair lead to significant adhesive layer cracking under low-speed impact. Psarras et al. [20] utilized finite element models to study the effects of stepped-scarf repair on the repair quality of composite material, finding that the performance of stepped-scarf repair is optimal when the number of steps equals the number of repair layers. Wang, Li, and Xie [21,22,23,24] studied the mechanical properties of ramped- and stepped-scarf repair structures through experiments and simulation. Several other scholars [25,26,27,28,29,30,31] have also conducted research and optimization on adhesive co-curing processes and scarf repair processes under different environmental conditions, and have verified their reliability through finite element analysis and experimental testing.

Currently, the research on composite material scarf repair mainly focuses on finite element theoretical analysis, while experimental studies on the effects of scarf repair process parameters on mechanical properties are limited [32]. Moreover, in the context of scarf repair, patches are predominantly fabricated using prepreg materials, which are not only costly but also incur high storage costs. Drawing on previous research, this paper introduces a mixed-scarf (MS) repair structure that amalgamates the features of both ramped- and stepped-scarf repair configurations. By utilizing fiber fabric as the patch material and implementing the vacuum-assisted resin-infusion (VARI) process for comprehensive one-time curing and repair, it is possible to enhance the efficiency of the repair process. Comparative analyses of the outcomes from various scarf structures were conducted, yielding substantial experimental evidence to support the repair of composite material damage.

## 2. Materials and Methods

### 2.1. Materials

Bisphenol A epoxy resin (EP51, the epoxy equivalent is 184 g/mol, the inorganic chlorine value is 7 mg/kg, and the viscosity is 13,483 mPa·s) was supplied by Nantong Xingchen Synthetic Materials Co., Ltd. (Nantong, China), The mechanical properties of the epoxy resin (E51) are presented in Table 1. The curing agent, triethylenetetramine (TA), was obtained from Sinopharm Chemical Reagent Co., Ltd. (Shanghai, China). The accelerator, 2,4,6-tri (dimethylaminomethyl) phenol (DMP30), which has an amine value of 600 KOH/g, was provided by Puyang Huicheng Chemical Materials Co., Ltd. (Puyang, China). The reactive diluent LS622 was sourced from Hubei Lvyuan Fine Chemical Co., Ltd. (Dangyang, China). Unidirectional carbon-fiber cloth T700 with a surface density of 200 g/m^2^ was supplied by Jiangsu Tianniao High-tech Co., Ltd. (Yixing, China).

### 2.2. Equipment

The electric hot-air oven (DZF-6050) was supplied by Beijing Hengtaifengke Experimental Equipment Co., Ltd. (Beijing, China). The vacuum-drying oven (DFZ-6090) was also provided by Beijing Hengtaifengke Experimental Equipment Co., Ltd. (Beijing, China). The composite-material vertical machining center (TV-510A, with an accuracy of 0.01 mm) was sourced from Hangzhou Youjia Precision Machinery Co., Ltd. (Hangzhou, China). The universal testing machine (CMT1104) was obtained from Zhuhai SANS Testing Technology Co., Ltd. (Zhuhai, China). The instrumented impact tester (HIT) was supplied by ZWICK GmbH (Ulm, Germany). The scanning electron microscope (Sigma300) was provided by Carl Zeiss AG (Oberkochen, Germany). The electronic balance (LQC-20002, with an accuracy of 0.01 g) was supplied by Kunshan Uniquevite Electronics Technology Co., Ltd. (Kunshan, China). The micrometer (DL9325, with an accuracy of 0.01 mm) was provided by Deli Group Co., Ltd. (Ningbo, China).

### 2.3. Sample Preparation

#### 2.3.1. Preparation of Composite Laminate

**Figure 1:**
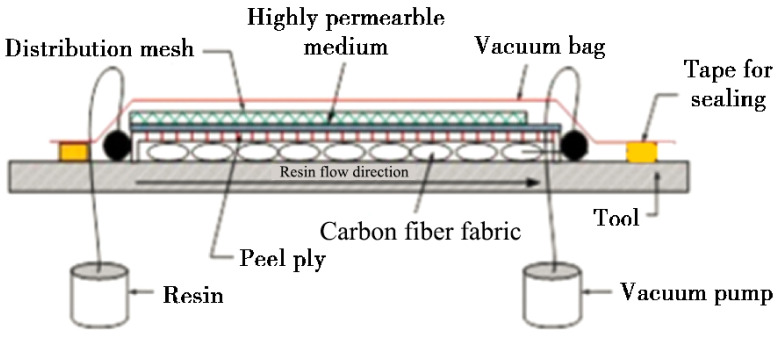
Preparation of composite laminate using VARI process.

The VARI process is depicted in Figure 1. The carbon-fiber unidirectional fabric (T700, areal density of 200 g/m^2^, dimensions 200 mm × 200 mm) comprises 24 plies arranged at a 0° orientation. Above the fabric layers, a peel ply, a high-permeability layer, and a distribution mesh are sequentially positioned. Subsequently, the air-tightness of the vacuum bag is verified to ensure proper sealing. The carbon-fiber fabric was pre-compacted by applying a vacuum at a pressure of 0.081 MPa.

At room temperature, the resin is injected through the resin inlet into the carbon-fiber preform. The resin flows along the high-permeability layer and infiltrates downward from the upper surface of the fiber layers. An electronic balance with an accuracy of 0.01 g is employed to ensure that the weight ratio of infused resin to fibers is maintained at precisely 1:1. After the completion of the resin-infusion process, the vacuum valve was closed to sustain a vacuum pressure of 0.081 MPa within the bag.

Subsequently, the mold is placed in an oven for heat curing at a temperature of 120 °C for a duration of two hours. Following this curing cycle, the mold is removed from the oven and allowed to cool naturally to room temperature.

The thickness of the laminate prepared using the VARI process was measured with a micrometer, which has an accuracy of 0.01 mm. The measurement yielded a thickness of 6.50 mm, accompanied by an error tolerance of ±0.02 mm. The dimensions of the carbon-fiber laminate were 200.00 mm × 200.00 mm × 6.50 mm.

#### 2.3.2. Preparation of Scarf-Repaired Specimens

**Figure 2:**
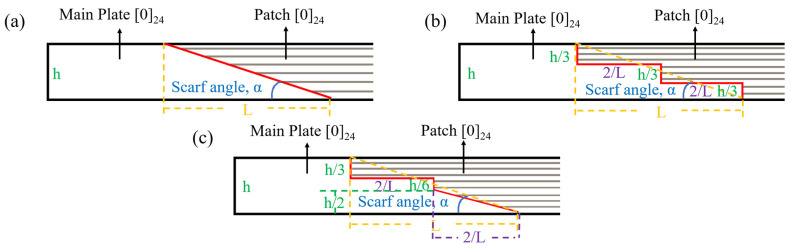
Scarf angle and dimensions schematic (**a**) RS, (**b**) SS, (**c**) MS.

Prefabricated damage was introduced into the composite laminate, with the damaged area measuring 25.00 mm in length. The affected region was subsequently sanded and cleaned. Following this, a repair structure for the damaged area was designed (as detailed in Table 2). Due to practical engineering constraints, it was necessary to avoid excessively small scarf angles; therefore, three specific angles were selected: 10°, 20°, and 30° (as illustrated in Figure 2, where the scarf angle refers to angle α between the line connecting the upper and lower endpoints of one side of the scarf repair area and the base surface).

As illustrated in Figure 2, the main plate thickness (h) is measured at 6.50 mm, and the bonding interface length (L) can be expressed as $L=\frac{h}{\tan(\alpha )}$. In the case of the RS repair structure, the ramp length is given by $\frac{h}{\sin(\alpha )}$. For the SS repair structure, the step height is defined as $\frac{h}{3}$, while the step length is represented by $\frac{L}{2}$. In terms of the MS repair structure, its upper section comprises two steps with heights of $\frac{h}{3}$ and $\frac{h}{6}$, respectively, along with a step length of $\frac{L}{2}$. The lower section features a ramp characterized by a height of $\frac{h}{2}$ and a corresponding length of $\frac{h}{2\cdot \sin(\alpha )}$.

**Figure 3:**
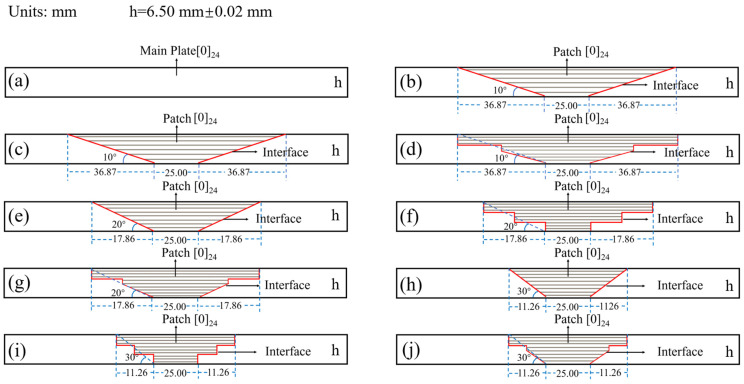
Schematic diagram of repair structure for composite laminate. (**a**) Main plate, (**b**) RS-10°, (**c**) SS-10°, (**d**) MS-10°, (**e**) RS-20°, (**f**) SS-20°, (**g**) MS-20°, (**h**) RS-30°, (**i**) SS-30°, (**j**) MS-30°.

As depicted in Figure 3, the machining of the damaged area was performed according to the specified scarf angles, step numbers, and sloped lengths using a composite-material vertical machining center (with an accuracy of ±0.01 mm). After machining, acetone was employed to clean the surface of the processed area in order to prepare an optimal substrate.

The repair patch utilized consisted of T700 carbon-fiber unidirectional fabric with an areal density of 200 g/m^2^ arranged in a [0]_24_ configuration. The shape and size of the patch are prepared according to Figure 2 and Figure 3. The prepared substrate and patch were then co-cured utilizing the VARI process (illustrated in Figure 1), adhering strictly to the curing procedures outlined in Section 2.3.1. During resin-infusion operations, an electronic balance (accuracy ± 0.01 g) ensured that the weight ratio of infused resin to fibers remained precisely at 1:1. Curing pressure was maintained at a constant level of approximately 0.081 MPa throughout this process resulting in a final scarf-repaired laminate.

**Figure 4:**
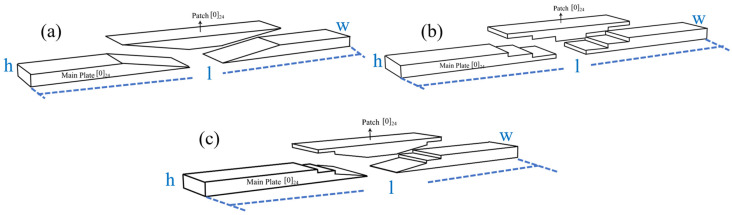
Schematic diagram of patch repair structure for composite laminate. (**a**) RS, (**b**) SS, (**c**) MS.

**Figure 5:**
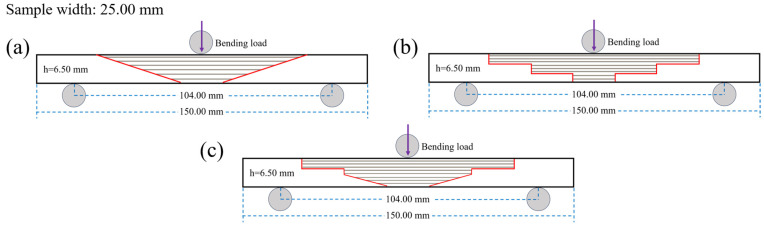
Schematic diagram of bending test. (**a**) RS, (**b**) SS, (**c**) MS.

**Figure 6:**
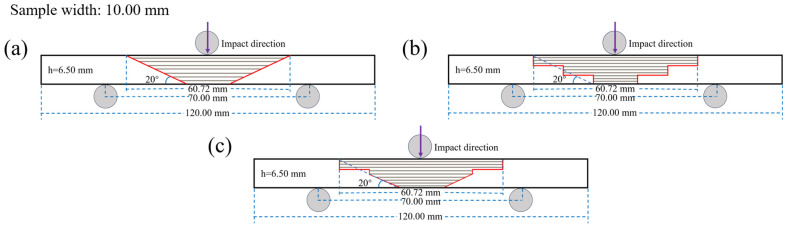
Schematic diagram of impact test. (**a**) RS-20°, (**b**) SS-20°, (**c**) MS-20°.

The thickness of this repaired laminate was measured using a micrometer (accuracy ± 0.01 mm), confirming its measurement as being exactly 6.50 mm with an error tolerance margin of ±0.02 mm. Subsequently, specimens were cut from this repaired laminate for bending and impact testing purposes (referenced in Figure 4, Figure 5 and Figure 6). Quality inspections were conducted following HB7224-95 standards [34] ensuring that all specimens exhibited no significant manufacturing defects.

### 2.4. Bending Test

Perform bending tests on all specimens with the scarf repair structures and main plate shown in Figure 3. A universal testing machine was employed to conduct three-point bending tests in accordance with the ASTM D790-2010 standard [35]. As illustrated in Figure 5, the dimensions of the bending specimens were 150.00 mm × 25.00 mm × 6.50 mm, with a span of 104.00 mm and a loading rate of 2 mm/min. A total of five sets of specimens were evaluated for each type of patch repair structure, and the mechanical property results were recorded as the average derived from these five sets of tests.

The recovery rates of the flexural properties are defined as the ratio of the flexural property recovery rates of the scarf repair structure to those of the main plate. That is,

$$\eta_{s}=\frac{\sigma_{f}^{s}}{\sigma_{f}^{m}}\times 100\%\;\mathrm{and}\;\eta_{m}=\frac{E_{f}^{s}}{E_{f}^{m}}\times 100\%$$

The parameter $\eta_{s}$ represents the recovery rate of flexural strength, where $\sigma_{f}^{m}$ denotes the flexural strength of the main plate and $\sigma_{f}^{s}$ indicates the flexural strength obtained from the scarf repair test. Additionally, $\eta_{m}$ signifies the recovery rate of flexural modulus, with $E_{f}^{m}$ referring to the flexural modulus of the main plate and $E_{f}^{s}$ representing the flexural modulus derived from the scarf repair test.

### 2.5. Impact Test

Instrumented impact tests were conducted on simply supported beams with a scarf angle of 20° for ramped-, stepped-, and mixed-scarf repair structures, in accordance with the ISO 179-2 standard [36]. As illustrated in Figure 6, the dimensions of the impact specimens were 120.00 mm × 10.00 mm × 6.50 mm, with a span of 70.00 mm. The striker specification was set at 2 J. A total of five sets of specimens were evaluated for each type of scarf repair structure, and the mechanical property results were recorded as the average derived from these five sets of tests.

### 2.6. Microstructure Characterization

Take the specimens that have experienced bending and impact damage, and utilize a composite-material vertical machining center (with an accuracy of 0.01 mm) to precisely cut the fractured sections (dimensions: 10.00 mm × 10.00 mm × h_f_, where h_f_ represents the thickness of the specimen following fracture failure). The damage morphology of the scarf repair structure was characterized using a scanning electron microscope (Sigma300, ZEISS, Germany). The fracture surfaces of the specimens were directly adhered to conductive adhesive (double-sided carbon conductive adhesive tape), and the observation areas were coated with gold to enhance conductivity.

## 3. Results and Discussion

### 3.1. The Flexural Properties of the Repaired Composite Laminate

#### 3.1.1. The Effect of Scarf Repair Structure on the Flexural Properties of Composite Laminate

**Figure 7:**
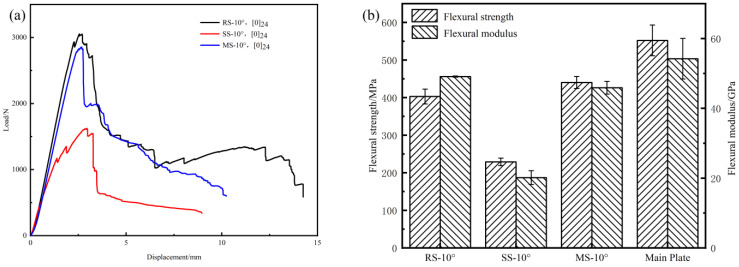

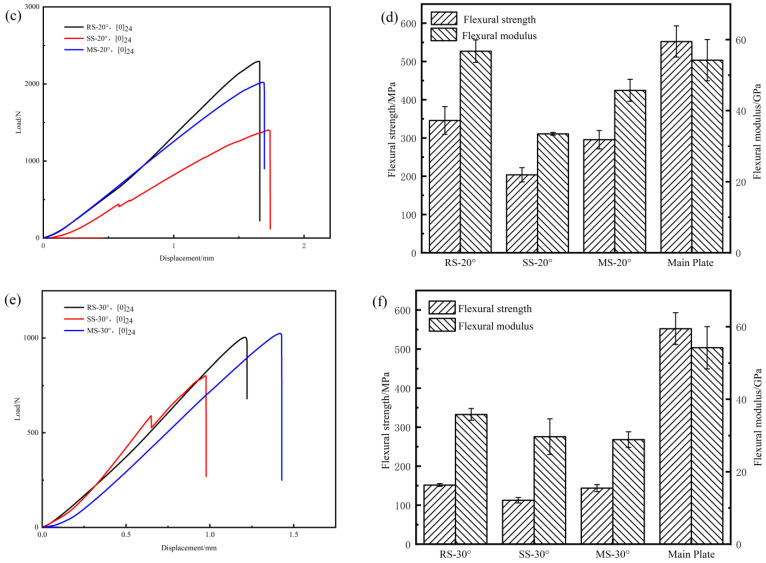
The flexural property of the three types of repaired structure. Load–deflection curves: (**a**) 10°-[0]_24_, (**c**) 20°-[0]_24_, (**e**) 30°-[0]_24_. Flexural strength and modulus: (**b**) 10°-[0]_24_, (**d**) 20°-[0]_24_, (**f**) 30°-[0]_24_.

The flexural properties of the three types of repaired composite structure are shown in Figure 7. From Figure 7a,c,e, it can be seen that among the three scarf repair structures, the ramped-scarf repair owns the highest flexural load. The maximum flexural loads of the mixed-scarf and ramped-scarf-repaired laminates are similar, and they exhibit similar load-deflection curves. The stepped-scarf-repaired laminate has the lowest flexural load.

As shown in Figure 7b,d,f, when the scarf angle is the same, the ramped-scarf repair has the best flexural properties, followed by the mixed-scarf repair, with the stepped-scarf repair showing the poorest performance. When the scarf angle is 10°and 20°, the flexural properties of the ramped- and mixed-scarf-repaired composites are significantly better than those of the stepped-scarf-repaired composites. Specifically, at a scarf angle of 10°, the flexural strength and modulus of the mixed-scarf-repaired composites are 92.18% and 128.11% higher than those of stepped-scarf-repaired composites. Compared to the ramped-scarf-repaired composites, the flexural strength and modulus of the mixed-scarf-repaired composites are slightly lower; the repair performance of the mixed-scarf repair composites becomes increasingly inferior to that of the ramped-scarf-repaired composites, especially as the scarf angle increases. This is because the stepped-scarf-repaired composites, due to the presence of right-angle steps, leads to stress concentration and lower interface bonding strength, resulting in poor performance. The ramped-scarf-repaired composites, for its favorable stress transition and uniform load distribution in interface, performs the best and can more effectively restore the strength and stability of the repaired structure. The mixed-scarf-repaired composites combines the advantages and disadvantages of both the ramped- and stepped-scarf-repaired composites, offering moderate repair performance. At smaller scarf angles, the mixed-scarf repair has a larger bonding area, resulting in performance similar to that of the ramped-scarf-repaired composites.

Table 3 presents the flexural property recovery rates of three types of scarf repair structure. The results indicate that the ramped-scarf-repaired structure exhibits the highest flexural property recovery rate. In comparison, the mixed-scarf repair demonstrates a slightly lower recovery rate than the ramped-scarf repair. However, it significantly surpasses that of the stepped-scarf repair. Notably, among these options, the mixed scarf with a scarf angle of 10° achieves the highest flexural strength recovery rate of 79.72%. The flexural strength recovery rate for mixed-scarf-repaired composites is higher by 6.77% and 38.24% compared to those of ramped- and stepped-scarf-repaired composites, respectively.

#### 3.1.2. The Effect of Scarf Angle on the Flexural Properties of Composite Laminate

**Figure 8:**
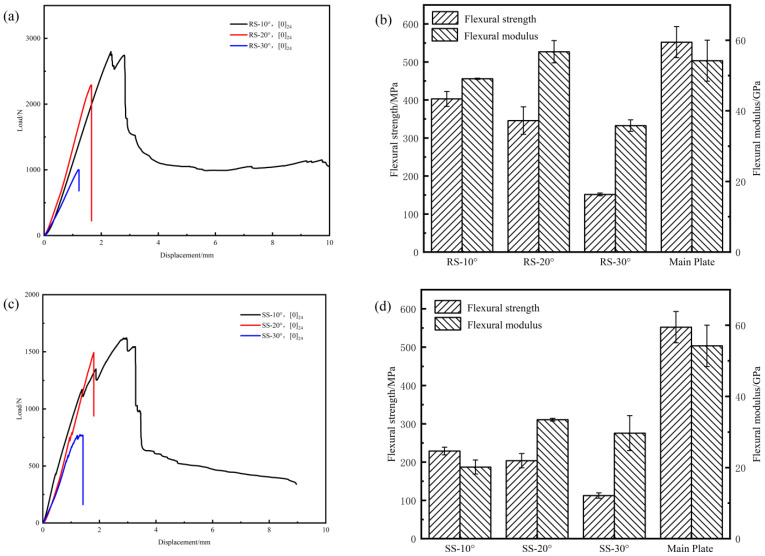

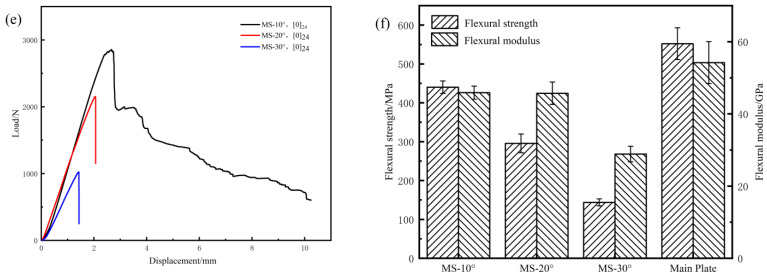
The flexural property of repaired composite laminate. Load–deflection curves: (**a**) RS-[0]_24_, (**c**) SS-[0]_24_, (**e**) MS-[0]_24_. Flexural strength and modulus: (**b**) RS-[0]_24_, (**d**) SS-[0]_24_, (**f**) MS-[0]_24_.

Figure 8 presents the flexural properties of the repaired composite with three different scarf angles. According to Figure 8a,c,e, it is observed that for a given scarf repair structure, the load decreases as the scarf angle increases. At a scarf angle of 10°, all three types of scarf repair structure demonstrate a significant drop of load maximum flexural load, followed by a gradual decline until complete bending failure. In contrast, at scarf angles of 20° and 30°, these structure experience sudden fracture and total failure upon attaining their flexural load. This behavior could be attributed to the larger bonding area of the repair structure at a scarf angle of 10°, which results in enhanced bonding strength. Conversely, at scarf angles of 20° and 30°, the decreased bonding area leads to diminished interfacial bonding strength, rendering the repair structure more vulnerable to abrupt fracture when subjected to flexural loads.

Figure 8b,d,f show that a decrease in the scarf angle corresponds to an increase in the flexural strength structure. Specifically, at a scarf angle of 10°, the flexural strength for the ramped-, stepped-, and mixed-scarf repair structures are measured at 402.88 MPa, 229.07 MPa, and 440.23 MPa, respectively. In contrast to the trends observed in flexural strength, an analysis of flexural modulus across various types of repair structure reveals that the highest values are attained at a scarf angle of 20°. At this particular angle, the flexural moduli for ramped-, stepped-, and mixed-scarf repairs are recorded as 56.74 GPa, 33.48 GPa, and 45.72 GPa, respectively. Furthermore, it is important to note that the flexural moduli of these repair structure at both scarf angles of 10° and 30° are lower than those observed at a scarf angle of 20°.

According to Table 3, as the scarf angle decreases, the flexural strength recovery rate increases. The mixed-scarf-repaired structure is particularly sensitive to variations in the scarf angle, as the scarf angle decreases from 30° to 10°, the flexural strength recovery rate increased to 53.68%. Notably, irrespective of the type of repair structure employed, the highest flexural modulus recovery rate is observed at a scarf angle of 20°.

### 3.2. The Effect of Scarf Repair Structure on the Impact Properties of Composite Laminate

**Figure 9:**
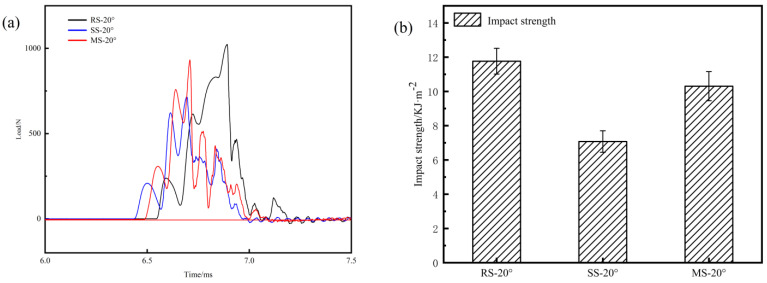
The impact property test results for the three types of composite laminate. (**a**) Load–time curves; (**b**) impact strength.

For a scarf angle of 20°, the impact property of the ramped-, stepped-, and mixed-scarf repair structures were examined, and the results are shown in Figure 9. From Figure 9a, it can be seen that the ramped-scarf repair structure has the highest impact load, reaching 1023 N, followed by the mixed-scarf repair structure with an impact load of 933 N, and subsequently, the stepped-scarf repair structure with an impact load of 711 N. The impact load curves show that all three repair structures exhibit peaks during the loading process to the maximum load. However, for the ramped-scarf repair structure, the first and fourth peaks are more prominent, while the second and third peaks are less noticeable. For the stepped- and mixed-scarf repair structures, the three peaks are all relatively prominent. This phenomenon can be attributed to the fact that the interfacial strength of the ramped-scarf repair structure is predominantly influenced by the interfacial bonding strength. In contrast, stepped- and mixed-scarf repair structures, which incorporate stepped features, also benefit from mechanical interlocking at their interfaces. This results in more pronounced peaks observed in the impact load curve.

From Figure 9b, it is evident that the impact performance results closely resemble those of the flexural performance. The ramped-scarf repair structure exhibits the highest impact strength, achieving a value of 11.77 KJ/m^2^. The mixed-scarf repair structure demonstrates a commendable second-best impact strength at 10.31 KJ/m^2^, which corresponds to 87.60% of the impact strength observed in the ramped-scarf repair structure. Conversely, the stepped-scarf repair structure records the lowest impact strength at 7.07 KJ/m^2^. Notably, the impact strength of the mixed-scarf repair structure is 45.83% greater than that of the stepped-scarf repair structure.

### 3.3. Failure Modes of Repaired Composite Laminate

The composite laminates were repaired through the design of a scarf repair structure. The bending and impact mechanical properties of the scarf repaired composite specimens were thoroughly analyzed. Given that the bonding interface represents a critical weak point in the mechanical performance of the scarf repair structure, it is essential to identify the failure modes of the specimens in order to effectively evaluate the performance of this bonding interface.

#### 3.3.1. Failure Modes Under Bending Load

**Figure 10:**
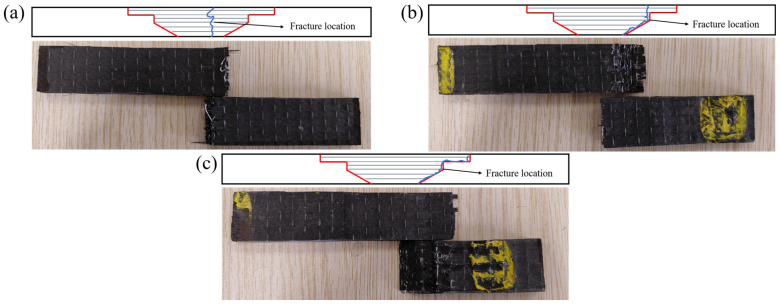
Typical bending failure modes of composite laminate. (**a**) Patch fracture; (**b**) mixed failure: patch fracture and adhesive layer cracking; (**c**) adhesive layer cracking.

Figure 10 illustrates the typical failure modes of the scarf repair structure subjected to bending loads. Through an analysis of the failure modes exhibited by both the scarf repair structure and the main plate under such loading conditions, we can categorize the actual failure modes of the scarf repair structure into three distinct types: patch fracture, mixed failure (patch fracture and adhesive layer cracking), and adhesive layer cracking.

Table 4 summarizes the failure modes of all scarf repair structures. For the main plate, when subjected to bending loads, matrix cracking initially occurs at the loading point, followed by fiber fracture. The side experiencing the bending load begins to deform under shear stress, and this deformation is subsequently transmitted to the opposite side until complete failure occurs (main plate fracture).

When the scarf angle is set at 10°, both mixed- and ramped-scarf repair structures exhibit the loading point that is positioned farther from the adhesive interface and possess a larger bonding area. Consequently, their failure mode resembles that of the main plate; damage initiates at the loading point and ultimately propagates to affect the backside of the specimen, culminating in complete failure (mode (a)). In contrast, for the stepped-scarf repair structure, significant stress concentration leads not only to patch fracture but also to adhesive interface failure (mode (b)).

At a scarf angle of 20°, ramped-, stepped-, and mixed-scarf repair structures demonstrate reduced bonding areas with closer proximity to the load center when subjected to bending loads. This configuration results in patch fractures and facilitates easy cracking at the bottom of the adhesive layer, leading to mixed failure (mode (b)).

Finally, an examination of a scarf angle of 30° reveals a significant reduction in the effectiveness of repair for ramped-, stepped-, and mixed-scarf repair structures. Under bending loads in this scenario, cracking initiates within the adhesive layer and progressively extends until total adhesive layer failure occurs (mode (c)).

**Figure 11:**
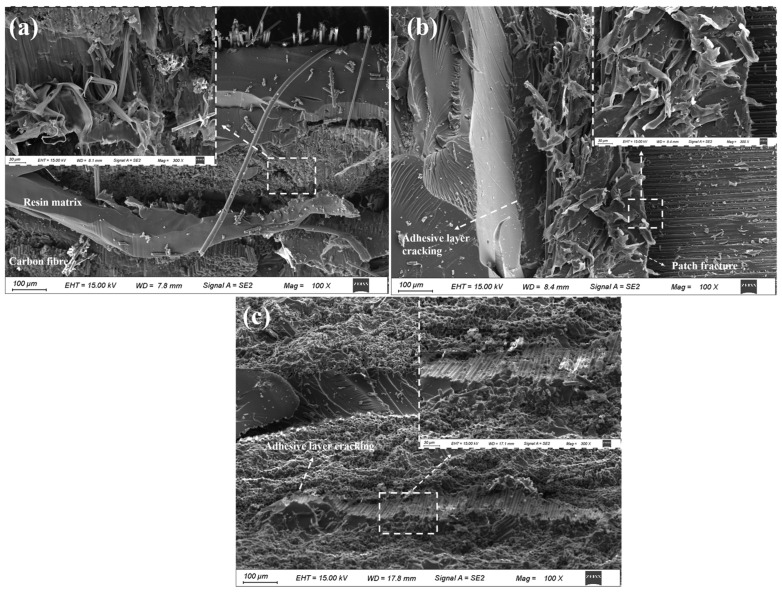
SEM images of typical bending failure modes of composite laminate. (**a**) Patch fracture; (**b**) mixed failure: patch fracture and adhesive layer cracking; (**c**) adhesive layer cracking.

The microscopic morphology of typical failure interfaces in scarf repair structure subjected to bending loads is illustrated in Figure 11. As observed in Figure 11, for mode (a), multiple failure modes—including tensile, compressive, bending, and interlaminar shear—manifest within the composite material under bending load. Consequently, the failure interface exhibits a range of damage characteristics, with its appearance indicative of patch fracture. This observation suggests that the interface of the repair structure corresponding to this mode demonstrates optimal performance, aligning with the results obtained from flexural tests.

For mode (b), under bending load conditions, patch fracture and adhesive interface failure emerge as predominant phenomena. In contrast, for mode (c), initial cracking occurs at the adhesive layer under bending load; this cracking progressively propagates until complete failure of the adhesive layer ensues.

#### 3.3.2. Failure Modes Under Impact Load

**Figure 12:**
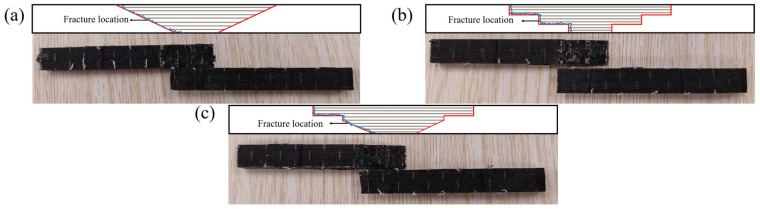
Impact failure modes of composite laminate. (**a**) RS-20°, (**b**) SS-20°, (**c**) MS-20°.

**Figure 13:**
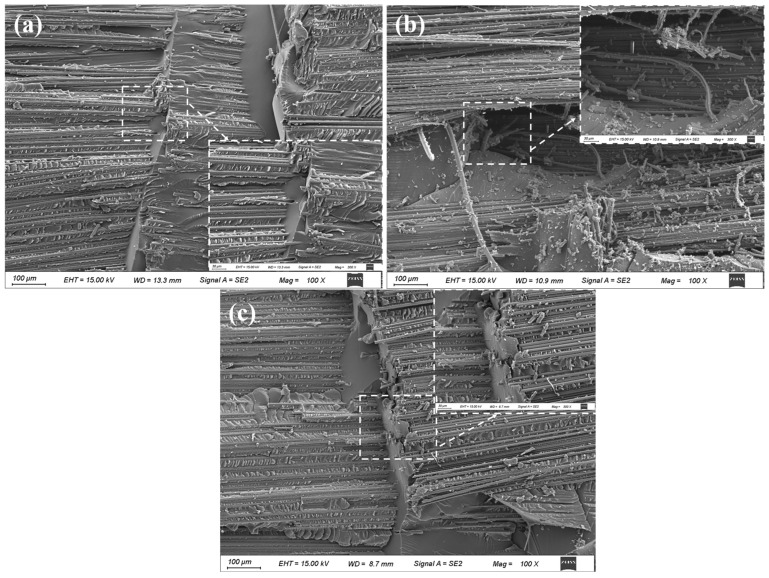
SEM images of impact failure modes of composite laminate. (**a**) RS-20°, (**b**) SS-20°, (**c**) MS-20°.

Figure 12 and Figure 13 are fracture surfaces for three types of scarf repair structure subjected to impact loading at a scarf angle of 20°. It was observed that all three types of scarf repair structure failed at the adhesive interface due to the relatively weak bonding strength. From Figure 13, it is evident that the adhesive interface in both ramped- and mixed-scarf repair structures are relatively flat, exhibiting strong adhesion between the resin and fibers without any noticeable defects. In contrast, the stepped-scarf repair structure presents an uneven surface within the repair area characterized by three right-angle steps. This geometric configuration hinders proper bonding between the resin and fibers, often resulting in insufficient fiber presence in these right-angle regions. Consequently, significant defects arise which affect the quality of the adhesive interface.

### 3.4. Discussion on Mechanical Properties and Adhesive Interface Quality of Scarf Repair Structures

It can be observed that for small scarf angles, the performance of mixed- and ramped-scarf repair structures is comparable. However, for larger scarf angles, the ramped-scarf repair structure exhibits superior mechanical properties. For identical scarf angles, the stepped-scarf repair structure demonstrates the least performance. The impact performance of these scarf repair structures parallels their flexural performance, the ramped-scarf repair structure achieves the highest impact performance while the stepped-scarf repair structure shows the lowest. Notably, the impact strength of mixed-scarf repair structure is approximately 87.60% that of the ramped-scarf repair structure; meanwhile, it is 45.83% greater than that of the stepped-scarf repair structure.

It is observed that for identical types of repair structure, the flexural strength decreases as the scarf angle increases. In terms of flexural modulus, irrespective of the type of repair structure employed, the maximum flexural modulus appears at a scarf angle of 20°. The mechanical properties of these repair structures are best at a scarf angle of 10°, followed by angles of 20° and 30°. At a scarf angle of 10°, the repair structure exhibits both the highest flexural strength and modulus recovery rates. Notably, a mixed-scarf repair structure demonstrates the greatest flexural-strength recovery rate, achieving 79.72%, which surpasses ramped- and stepped-scarf repair structures of 6.77% and 38.24%, respectively.

At a consistent scarf angle, the ramped-scarf repair structure exhibits the most favorable adhesive interface, followed by the mixed- and stepped-scarf repair structures. When considering a scarf angle of 10°, both ramped- and mixed-scarf repair structures demonstrate superior adhesive interface quality, with their predominant flexural failure mode being patch fracture, which closely resembles that of the main plate. In contrast, at a scarf angle of 20°, the primary impact failure mode is characterized by adhesive interface fracture. Here, ramped- and mixed-scarf repair structures maintain comparable adhesive interfaces; however, significant defects are observed at the interface in stepped-scarf repair structure.

## 4. Conclusions

From the perspective of scarf repair structures: the impact performance of scarf repair structures is closely related to their flexural strength. Both parameters indicate that ramped-scarf and mixed-scarf repair structures demonstrate comparable effectiveness, which surpasses that of stepped-scarf repair structures.

From the perspective of scarf angle: The mechanical properties of the scarf repair structure are optimized at a scarf angle of 10°, followed by angles of 20° and 30°. Under identical types of scarf repair structures, it is observed that flexural strength decreases as the scarf angle increases. However, with regard to flexural modulus, irrespective of the type of scarf repair structure employed, the highest flexural modulus is attained at a scarf angle of 20°.

From the perspective of the adhesive interface: For a given scarf angle, the ramped-scarf repair structure demonstrates superior adhesive interface quality, followed by the mixed-scarf and stepped-scarf repair structures. Notably, the adhesive interface quality of the scarf repair structure is optimized at a scarf angle of 10°.

In summary, the ramped- and mixed-scarf repair structures exhibit comparable mechanical performance and demonstrate superior adhesive interface quality, significantly surpassing that of the stepped-scarf repair structure. The mixed-scarf repair structure is particularly suitable for small-angle repairs, whereas the ramped-scarf repair structure is more appropriate for larger angles.

## Figures and Tables

**Table 1:** Mechanical properties of epoxy resin (E51) casting specimens [33].

Mechanical Properties	Tensile Strength/MPa	Tensile Modulus/GPa	Fracture Elongation/(%)	Flexural Strength/MPa	Flexural Modulus/GPa	Compressive Strength/MPa	Impact Strength/(J·m^−2^)
Epoxy resin (E51)	47.30	3.57	1.40	62.74	3.43	101.08	9.08 × 10^3^

**Table 2:** Test specimens of scarf-repaired composites.

Sample Number	Sample Type	Main Plate Lay-Up Method	Patch Lay-Up Method	Scarf Angle α	Number of Steps	Number of Ramps	Objective
Main Plate	Main plate	[0]_24_	---	---	---	---	Reference
RS-10°	Ramped scarf	[0]_24_	[0]_24_	10°	---	1	Compare
SS-10°	Stepped scarf	[0]_24_	[0]_24_	10°	3	---	Compare
MS-10°	Mixed scarf	[0]_24_	[0]_24_	10°	2	1	Compare
RS-20°	Ramped scarf	[0]_24_	[0]_24_	20°	---	1	Compare
SS-20°	Stepped scarf	[0]_24_	[0]_24_	20°	3	---	Compare
MS-20°	Mixed scarf	[0]_24_	[0]_24_	20°	2	1	Compare
RS-30°	Ramped scarf	[0]_24_	[0]_24_	30°	---	1	Compare
SS-30°	Stepped scarf	[0]_24_	[0]_24_	30°	3	---	Compare
MS-30°	Mixed scarf	[0]_24_	[0]_24_	30°	2	1	Compare

**Table 3:** Flexural property recovery rates of scarf repair structure.

Sample Number	Main Plate	RS-10°	RS-20°	RS-30°	SS-10°	SS-20°	SS-30°	MS-10°	MS-20°	MS-30°
Flexural strength recovery rate (%)	——	72.95	62.63	27.46	41.48	36.90	20.41	79.72	53.51	26.04
Flexural modulus recovery rate (%)	——	90.57	104.66	66.09	37.17	61.75	54.77	84.67	84.33	53.26

**Table 4:** Failure modes of composite laminate under bending load.

Sample Number	Main Plate	RS-10°	RS-20°	RS-30°	SS-10°	SS-20°	SS-30°	MS-10°	MS-20°	MS-30°
Failure modes	Main plate fracture	Mode (a)	Mode (b)	Mode (c)	Mode (b)	Mode (b)	Mode (c)	Mode (a)	Mode (b)	Mode (c)

## Data Availability

The original contributions presented in the study are included in the article, further inquiries can be directed to the corresponding author.

## Abbreviations

The following abbreviations are used in this manuscript:

RS	Ramped Scarf
SS	Stepped Scarf
MS	Mixed Scarf
VARI	Vacuum-assisted resin infusion
